# Explicit Oral Narrative Intervention for Students with Williams Syndrome

**DOI:** 10.3389/fpsyg.2017.02337

**Published:** 2018-01-15

**Authors:** Eliseo Diez-Itza, Verónica Martínez, Vanesa Pérez, Maite Fernández-Urquiza

**Affiliations:** ^1^LOGIN Research Group, University of Oviedo, Oviedo, Spain; ^2^SUIGC, University School Gimbernat-Cantabria, Torrelavega, Spain

**Keywords:** Williams syndrome, pragmatic impairment, oral narrative, effective intervention, language development, narrative intervention, neurodevelopmental disorders, at risk of school failure

## Abstract

Narrative skills play a crucial role in organizing experience, facilitating social interaction and building academic discourse and literacy. They are at the interface of cognitive, social, and linguistic abilities related to school engagement. Despite their relative strengths in social and grammatical skills, students with Williams syndrome (WS) do not show parallel cognitive and pragmatic performance in narrative generation tasks. The aim of the present study was to assess retelling of a TV cartoon tale and the effect of an individualized explicit instruction of the narrative structure. Participants included eight students with WS who attended different special education levels. Narratives were elicited in two sessions (pre and post intervention), and were transcribed, coded and analyzed using the tools of the CHILDES Project. Narratives were coded for productivity and complexity at the microstructure and macrostructure levels. Microstructure productivity (i.e., length of narratives) included number of utterances, clauses, and tokens. Microstructure complexity included mean length of utterances, lexical diversity and use of discourse markers as cohesive devices. Narrative macrostructure was assessed for textual coherence through the Pragmatic Evaluation Protocol for Speech Corpora (PREP-CORP). Macrostructure productivity and complexity included, respectively, the recall and sequential order of scenarios, episodes, events and characters. A total of four intervention sessions, lasting approximately 20 min, were delivered individually once a week. This brief intervention addressed explicit instruction about the narrative structure and the use of specific discourse markers to improve cohesion of story retellings. Intervention strategies included verbal scaffolding and modeling, conversational context for retelling the story and visual support with pictures printed from the cartoon. Results showed significant changes in WS students’ retelling of the story, both at macro- and microstructure levels, when assessed following a 2-week interval. Outcomes were better in microstructure than in macrostructure, where sequential order (i.e., complexity) did not show significant improvement. These findings are consistent with previous research supporting the use of explicit oral narrative intervention with participants who are at risk of school failure due to communication impairments. Discussion focuses on how assessment and explicit instruction of narrative skills might contribute to effective intervention programs enhancing school engagement in WS students.

## Introduction

Williams syndrome (WS) is a neurodevelopmental genetic disorder which affects an estimated 1 in 7,500 to 10,000 people. It is caused by a deletion of 26 to 28 genes from a specific region on one copy of chromosome 7 (7q11.23). It is characterized by medical problems and mild to moderate intellectual disability and learning problems. In a seminal study, the distinctive cognitive profiles of three adolescents with WS were presented as cases of dissociation between language and cognitive functions (Bellugi et al., 1988). Claims of intactness or selective sparing of language in WS were later challenged by research with individuals speaking Italian, French and Spanish (Volterra et al., 1996; Karmiloff-Smith et al., 1997; Diez-Itza et al., 1998).

Further programs of research of the neurocognitive abilities of children and adults with WS described a specific, uneven profile with peaks and valleys, reflecting dissociations within and across cognitive domains. In this unusual pattern of strengths and weaknesses, language and face recognition were considered relatively spared when compared to visuospatial construction (Bellugi et al., 2000; Mervis et al., 2000). Nevertheless, there is strong evidence of complex interdependence between language and cognitive abilities in school-age children and adults with WS, which is not consistent with the claim for excellent language abilities in the WS population (Mervis, 1999; Mervis et al., 2004; Mervis and Becerra, 2007).

From a developmental point of view, the fractionation of the phenotypical outcomes observed in WS is interpreted as the result of complex and differential trajectories of development from the outset (Karmiloff-Smith, 1998). Such an approach allows for a dynamic interpretation of cognitive and behavioral outcomes in neurodevelopmental genetic disorders involving transactions with the environment at all levels over ontogenetic time (Mervis and Klein-Tasman, 2000; Karmiloff-Smith, 2011). A central assumption is that profiles are potentially modifiable by specific types of environmental inputs such as explicit interventions (Fidler et al., 2011). Using broad assessment and targeted intervention based on prior in-depth syndrome-specific research might then be effective in enhancing protective factors and reducing risk factors in the development of individuals with WS (D’Souza and Karmiloff-Smith, 2016).

Very few studies have assessed cognitive development of individuals with WS longitudinally (Mervis et al., 2012). Only one of them addressed the progress in educational attainment, finding a lack of improvement in academic skills but not a decline in IQ, and concluding the need for interventions focusing on daily language and communication skills (Udwin et al., 1996). A stereotyped description of WS, portraying its profile as showing near-normal language and social skills, has often led to discontinuation of language intervention once the child’s speech is fluent. However, despite accelerated development after a delay in language onset, pragmatics remain impaired in WS throughout the school years (Mervis and John, 2010; Mervis and Velleman, 2011). Pragmatic impairment in students with WS involves an additional risk factor for school failure as it may account for some of the difficulties in school engagement. Together with the atypical social phenotype, it may contribute to social vulnerability at school (Jawaid et al., 2012).

### Vulnerability and Social Cognition in WS

Social vulnerability and higher rates of social victimization are common in individuals with developmental disorders (Fisher et al., 2013). Atypicalities in social cognition may contribute to social vulnerability in these populations and may increase the risk of social isolation, bullying, and overall unsteady relationships in their social environments. Individuals with WS tend to show indiscriminate approachability, intense gazing, anxiety, distractibility, along with inappropriate and excessive chatter and social evaluation (Jawaid et al., 2012). This atypical social profile may explain why students with WS have difficulty maintaining peer relationships, despite their unusually friendly and social nature (Bellugi et al., 1999; Bellugi et al., 2007; Järvinen-Pasley et al., 2008).

Children with WS and Autism (ASD) have been described as the extremes of a continuum in terms of social cognition (Reilly et al., 1990; Jones et al., 2000). However, recent studies have also pointed out subtle similarities between ASD and WS concerning a number of difficulties in social interaction and pragmatic skills (Brock et al., 2009; Lacroix et al., 2016). Pragmatic assessment and intervention with these populations is recommended to enhance communicative skills necessary for school engagement (Philofsky et al., 2007).

### Pragmatic Development in WS

Research on pragmatic development focuses on how children acquire the knowledge for the appropriate and effective use of language in interpersonal situations (Ninio and Snow, 1996). Mastery of appropriate speech use depends on cognitive and social skills. Thus, neurodevelopmental disabilities in students with WS may affect pragmatic development, i.e., the acquisition of conversation and discourse skills, including narrative abilities.

#### Pragmatic Conversation Skills in WS

The WS population was early characterized as showing ease to engage in conversation and to accept responsibility for maintaining the interaction (Reilly et al., 1990). However, later research has pointed out that their conversational exchanges tend to be inappropriate and superficial. For example, they might reverse the role in interviews, asking personal questions to the researchers (Lacroix et al., 2007; Järvinen-Pasley et al., 2008). Parent and teacher reports signal inappropriate initiations of conversation and use of stereotyped language (Laws and Bishop, 2004; Philofsky et al., 2007). Qualitative analysis of the conversation skills confirm the existence of pragmatic anomalies against the initial impression that endorses individuals with WS for being good at maintaining conversational flow (Brock, 2007; Mervis and Becerra, 2007; Lacroix et al., 2016).

Children and adolescents with WS produced fewer utterances in collaborative conversation and less often satisfied other’s requests compared to mental age-matched TD children (Lacroix et al., 2007). In a pilot study, they were found to provide too little information for the conversational partner in the context of high levels of conversational inadequacy (Stojanovik et al., 2001). Systematic conversational analysis showed that children with WS produced fewer continuations compared to SLI and TD control groups, so their speech was characterized as being heavily ‘parasitic’ on the interlocutor’s contributions. They provided insufficient information as well as a higher number of inadequate responses to requests for information and clarification, and they showed significantly more difficulties with interpreting meaning, either literal or inferential (Stojanovik, 2006). In contrast, the case study of a child with WS suggested that impressions of linguistic competence may be the result of compensatory conversational strategies, such as the awareness of conversational partner’s interactive needs and the attentiveness to their affective state. Good interactional skills were reported in areas such as turn-taking, turn maintenance, topic management and conversational repair, so that the conversation flows easily, giving an impression of relevance and control (Tarling et al., 2006).

Developmental delays in communicative intentionality and social cognitive skills, including theory of mind abilities, have also been reported (Tager-Flusberg and Sullivan, 2000; Laing et al., 2002). Using an experimental paradigm, Asada et al. (2010a,b) found that children with WS produced fewer communication repairs than TD children when they were verbally misunderstood and they did not verbalize more when they were not attended to than when they were, thus showing an atypical interactional behavior. These results were interpreted as children with WS having a strong motive to interact with others but little motive to share what they meant, which is highly suggestive of theory of mind deficits. In a referential communication task, children with WS showed more non-verbal clarification requests (i.e., pointing gestures and puzzled gazes) than TD children and poorer abilities to use contextual information during ambiguous reference resolution. This was interpreted as a consequence of overall impairments in attention monitoring, visual search, inferring communicative intentions, as well as interpreting verbal messages (Skwerer et al., 2013). Early joint attention problems and limitations in secondary intersubjectivity may be the basis of later pragmatic difficulties (Laing et al., 2002; Mervis et al., 2003). Longitudinal research found that deficits at ages 9–12 years in the ability to verbally extend information were predicted by pragmatic abilities in triadic interactions at age 4 (John et al., 2012).

#### Pragmatic Narrative Skills in WS

Pragmatic development involves the ability to produce extended discourse and genre-specific forms as a major achievement of language learning. Extended discourse emerges from conversation both interactively and developmentally (Ninio and Snow, 1996). Conversationally embedded stretches of discourse free themselves and children develop a new level of organization of speech: the comprehension and production of narratives, which are considered a universal, basic mode of thought (Bruner, 1986, 1991; Engel, 1995).

Picture-book narration has traditionally been employed to study the development of narrative skills, being considered a natural setting that mirrors the mother–child interaction format of book reading. Bamberg (1987) introduced a method of narrative research based on the wordless picture-book “Frog, where are you?” (Mayer, 1969), pointing out that it allowed for the assessment of narrative development at very early stages, providing data of natural discourse rich enough to be analyzed at the microstructural linguistic level as well as at the macrostructural level of discourse organization. Within the “Frog story” (FS) paradigm, typical and atypical narrative development has been extensively studied cross-linguistically and throughout the school years and adulthood (Berman and Slobin, 1987, 1994; Berman, 1988).

Concerning the microstructural and the macrostructural aspects of narrative discourse, the narrative skills of children and adolescents with WS have been characterized as proficient when compared to clinical populations of the same cognitive level. Reilly et al. (1990) conducted the first study of narrative skills of four adolescents with WS, using the FS. When compared to a Down syndrome (DS) control group, they generated narratives with more grammatical complexity and structural coherence, showing an excessive use of affective and evaluative devices (i.e., character voice, intensifiers, exclamations, sound effects and rhetorical questions). They concluded that, as a characteristic of WS, is the use of a charming, although anomalous, affective expressivity when retelling a narrative. In a larger study also using the FS, younger children with WS generated narratives with more morphological errors and less complex syntax than those of TD age- and gender-matched children, but with a wider range of evaluative devices. Differences in structural linguistic abilities were explained as a consequence of the linguistic and cognitive impairments while differences in the use of engaging devices were considered a reflection of “excessive sociability” of children with WS (Losh et al., 2000).

The role of language vs. intellectual impairment in narrative production of the FS was investigated comparing school-age children with WS to paired SLI and TD children. Although WS children generated narratives of a similar length than those from TD children, their narratives presented more morphological errors and less frequency of complex sentences, showing a similar morphosyntactic profile to SLI children. However, they scored lower than TD and SLI children on macrostructural narrative measures, failing to integrate the characters and episodes in the thematic structure of the story and tending to focus on elaborated descriptions of specific episodes. Only the use of evaluative devices was considered a relative strength of the WS group. Results were interpreted in terms of a dissociation between the development of linguistic forms and the pragmatic ability to use them in order to build up integrated narratives (Reilly et al., 2004).

Cross-linguistic research with the FS confirmed the atypical narrative profile of WS. American, French, and Italian school-age children and adolescents with WS presented an excessive use of social evaluations during storytelling when compared to TD peers (Reilly et al., 2005). French-speaking WS children and adolescents also performed over DS controls but under TD chronological age (CA)-matched peers in the number of utterances and story-schema elaboration (Lacroix et al., 2007). Narratives of Spanish and Portuguese adolescents and young adults with WS showed low coherence at the local and global levels, lacking integration and inferencing. They tended to lose the main thread of the story and presented a limited use of cohesive markers and an excessive use of evaluative devices (Garayzábal Heinze et al., 2007). They showed low levels of structural coherence and complexity, and moderate levels of content diversity and emotional commitment with the storytelling, relying on diversity of narrative content at the expense of narrative coherence (Gonçalves et al., 2010). In a longitudinal single-case study, a young adult with WS, after an intervention devised to promote a number of linguistic and cognitive abilities, maintained the reference to affective states along with the use of evaluative devices, but failed to improve the production of cognitive inferences necessary to build up the narrative coherence (Fernández-Prieto et al., 2011).

Using single pictures and picture story sequences, Marini et al. (2010) assessed the narrative abilities of Italian-speaking children, adolescents and young adults with WS. They showed mental-age performance at the microstructural level (i.e., phonological, lexical, and morphosyntactic skills), but their narratives were less informative as well as less coherent on the local and global levels than those produced by the TD group, especially when generating a story upon the picture sequences. Results were interpreted in terms of a selective impairment in macrolinguistic (i.e., discourse-level) processing in WS. Van Den Heuvel et al. (2016) compared the developmental courses of structural and pragmatic language skills in Dutch school-aged children with WS to children with idiopathic intellectual disability (IID). Narrative ability was assessed using the Bus Story Test (Renfrew, 1997). Children with WS showed diverging developmental trajectories across language domains with increasing variability. They produced fewer utterances containing core information, and more unrelated and noise utterances compared to children with IID. Irrelevant and off- topic extraneous information was considered a syndrome-specific characteristic of WS. Based on a silent film adapted from a picture book of the “Frog story” series, we examined the narrative coherence and cohesion of Spanish-speaking adults with WS. Recall and sequential order of scenarios, episodes, and events were assessed together with the use of discourse markers. It was concluded that narrative competence in WS may be more impaired in terms of macrostructural organization of discourse than in terms of linguistic cohesion (Diez-Itza et al., 2016).

Overall, these studies underscore the non-homogeneous character of the conversational and narrative skills of children, adolescents and adults with WS. Despite their strengths in formal language and their sociability, they present pragmatic problems that limit their ability to participate in and benefit from educational opportunities. Therefore, recommendations for intervention for school-age children with WS include focus on pragmatic skills as critical for both academic performance and peer relationships (Mervis and John, 2010; Mervis and Velleman, 2011). Narratives are the natural context for such language skills to develop, and children who are competent at narration tend to do well in school (Griffin et al., 2004). Thus, narrative language skills have been considered an important target of assessment and intervention from the early years, and the narrative-primacy view has greatly influenced curricular practice for early literacy training (Hemphill and Snow, 1996).

### Narrative Intervention

In the absence of valid formal assessments, narratives provide very relevant and natural samples of pragmatic language skills as they require the ability of bridging cognitive, linguistic, and social domains. Storytelling abilities are good predictors of learning and literacy difficulties contributing to academic failure. Children with and without language impairment can learn complex language and narrative structure skills through minimal but high-quality explicit narrative language intervention (Spencer and Slocum, 2010; Spencer et al., 2015; Petersen and Spencer, 2016). Narrative intervention provides a flexible framework for dynamic assessment and progress monitoring within “Response to Intervention” (RTI) methods, which intend to go beyond “wait to fail” models in designing early intervention for children at risk of school failure (Petersen and Spencer, 2014).

Narrative assessment with diverse methodologies focuses on measurements of microstructure linguistic features (i.e., vocabulary, morphology, and syntax primarily at the sentence level), and macrostructure elements of the narratives (i.e., content, organization, and overall quality at the discourse level) (Peterson and McCabe, 1983; McCabe and Rollins, 1994; Bliss et al., 1998; McCabe et al., 2008; Heilmann et al., 2010; Petersen and Spencer, 2012).

There is relatively little research on narrative language profiles of children and adolescents with developmental disabilities (Finestack, 2012). Empirical evidence draws on research of children and adolescents with DS, Fragile X syndrome (FXS), Autistic Spectrum Disorder (ASD), WS, and Specific Language Impairment (SLI). Although children with DS and FXS show impairments both at the microstructure and the macrostructure levels of the narratives, macrostructure narrative skills may develop as relative strengths in both populations (Boudreau and Chapman, 2000; Finestack et al., 2012; Channell et al., 2015). Individuals with ASD display difficulties in microstructure language measures and in the use of cohesive and evaluative devices (King et al., 2013). Narratives of children with ASD have been linked to theory of mind and conversational competence, and have been reported to be simplistic from a macrostructural point of view, including odd tangential comments about the story, and lacking causal coherence and organization (Capps et al., 2000; Norbury et al., 2014; Gillam et al., 2015). School-age children with SLI produced poorer narratives both at the microstructure and macrostructure levels compared to TD peers (Fey et al., 2004; Marini et al., 2008). Children with SLI and WS exhibited similar morphosyntactic performances, although the WS group presented fewer story components and less thematic integration than the SLI group (Reilly et al., 2004).

These findings suggest that children and adolescents with developmental disabilities may benefit from narrative intervention targeting both microstructure and macrostructure levels. Ukrainetz (2006) proposed narratives as a context for teaching students with language impairments the language needed for academic success. This “Contextualized Language Intervention” approach proposes the use of specific teaching steps to scaffold explicit semantic, syntactic and pragmatic language skills. For younger students, the ultimate objective is to promote the moving from a conversational context for storytelling to independent narrative retelling. For older students, intervention focuses on narrative structure, cohesion, and story creation. A contextualized approach for children with language impairment yielded better clinical outcomes than a decontextualized language intervention both in sentence-level measures and in a general measure of narrative language ability. The effect was moderately large on narrative comprehension and narrative microstructure but small on the macrostructure (Gillam et al., 2012).

In a review of three decades of research, Petersen (2011) reported only nine studies evaluating narrative interventions delivered to school-age children with language impairment (aged 3–21). Although results varied depending on the design of the research, significant gains were reported both for narrative microstructure and macrostructure as an effect of narrative intervention with preschool- and school-age children with delayed and impaired language development. Children improved the quality of storytelling, and consequently their ability to participate in and benefit from mainstream classroom activities (Davies et al., 2004; Swanson et al., 2005).

However, evidence of the impact of narrative intervention on populations with developmental disabilities is even scarcer, with no studies on WS. Preschoolers with developmental disabilities exhibited gains in comprehension and production of narratives after a short intervention based on *Story Champs*, a specific curriculum for teaching children narrative skills (Spencer et al., 2013). Individualized narrative interventions for school-age children with ASD based on repeated retellings, script-frameworks, and microstructure and macrostructure explicit instruction proved its efficacy on improving story complexity, story structure, and the use of mental state and causal language (Petersen et al., 2014; Gillam et al., 2015; Hilvert et al., 2016).

Beyond cultural differences, researchers point out the need for effective, targeted interventions to promote independence and to enhance communication and social functioning in students with WS (Järvinen-Pasley et al., 2008; Jawaid et al., 2012; Ji et al., 2014). However, there is a great disproportion between the extensive basic research of WS and the limited applied intervention research of this population. Given the current level of knowledge of the behavioral phenotype of WS, the start of research focusing on the development and evaluation of methods of intervention has been considered a vital effort (Mervis and John, 2010).

There is a need to examine the types of intervention that may be the most beneficial to individuals with WS as there is a lack of evidence about effective interventions focusing on areas of vulnerabilty. Semel and Rosner (2003) authored one of the first comprehensive analyses of the research literature, aiming at providing syndrome-specific intervention and innovative techniques for developing the potential of individuals with WS. They consider the ability to engage in meaningful discourse and produce interesting stories the “pièce de résistance” of expressive language for individuals with WS, and suggest interventions based on those strengths to facilitate discourse and to improve narrative skills.

It has been suggested that storytelling could provide an optimal context for scaffolding skills such as event sequencing or perspective taking, along with the linguistic tools necessary to express the key story elements (Channell et al., 2015). Research-supported principles regarding difficulties in narrative language, strengths in narrative macrostructure, evidence for the impact of interventions, and effects of visual support and narrative tasks have been proposed to design and implement narrative language intervention for children and adolescents with developmental disabilities (Finestack, 2012). Thus, narrative intervention focused on oral storytelling skills could help students with WS in meeting academic requirements, enhancing school engagement and providing a contribution to their academic-social environment.

## Objectives

Students with WS might have relative strengths in grammatical and lexical aspects of language production, but these linguistic skills usually do not correspond to pragmatic abilities necessary for effective communication. This pragmatic impairment observed in school-age individuals with WS results in a limited capacity to build extended discourse in order to relate personal or fictional events in everyday conversational settings. Thus, despite showing remarkable linguistic abilities and a highly social and empathetic behavioral phenotype, limitations in pragmatic narrative ability may account for students with WS struggling to maintain social relations and to benefit from school inclusion to avoid academic failure.

Explicit oral narrative assessment and intervention has proven effectiveness to preventing academic failure and enhancing school achievement in typically and atypically developing students of all ages. Narrative competence has been assessed only to a limited extent in individuals with WS but, to our knowledge, there are no results about possible effects of narrative intervention with this population. Thus, the aims of the present study were:

(1) To assess narrative competence of a group of students with WS of different ages based on their ability to generate and retell oral narratives from a silent film, using linguistic measures of microstructural and macrostructural productivity and complexity.
(1) To assess the feasibility and effects of an individual explicit oral narrative intervention for the group of students with WS. A short semi-manualized intervention (four sessions) was delivered based on repeated generation and retelling of the story, with visual support and immediate scaffolding from an interventionist. Effects of intervention on narrative microstructure and macrostructure were evaluated.

## Materials and Methods

### Participants

Eight students with WS (four males, four females) from monolingual Spanish-speaking families were drawn from a larger research project on cross-syndrome linguistic comparisons (Diez-Itza et al., 2014). However, the assessment and intervention reported in this paper had not been previously conducted. Their mean CA was 16;8 (range: 8;11–24;04). All the participants had been previously diagnosed with WS using the FISH test (Fluorescence *In Situ* Hybridization) and presented the typical clinical phenotype. They were attending different levels of school in Spain: mainstream primary schools (3), special schools (2), and special vocational education centers (3).

The participants had been matched in previous studies to different samples of 5-year-old typically developing children on the basis of MLU as an indicator of verbal age. In one study of spontaneous conversation (Diez-Itza et al., 2017) the TD group had a mean age of 5;5 (range: 5;0–5;11), and a mean MLUw of 4,8 (range: 2;6–9;0). In another study of narratives in conversation (Shiro et al., 2016), the TD group had a mean age of 5;8 (range: 5;4–6;5), and a mean MLUw of 6,6 (range: 4;7–10;3). Thus, verbal age for the students with WS in the present study corresponds to that of TD children in the last year of preschool in the Spanish educational system (mean age: 5;7; range: 5;5–6;5). Consequently, it was considered that in all cases the participants with WS would have a sufficient level of linguistic skills to avoid floor effects at pretest assessment. Furthermore, they had no physical impairments that would interfere with the ability to perform the narrative tasks during the intervention. In order to control for non-verbal intellectual levels, the performance scales of the WISC-R and WAIS-III (Wechsler, 1999a,b) were administered to the participants at pretest (Mean PIQ: 64; range: 44–90).

Approval for human subjects research was granted by the research ethics board of the affiliated university, and written consent was obtained from the parents/guardians of all participants.

### Procedure

#### Narrative Task

Oral narratives were elicited individually from a 6-min silent episode of the Tom and Jerry cartoon series (“The Puppy Tale”). The same procedure was repeated at pretest (Time 1) and posttest (Time 2). Each subject watched the film in a quiet room, only accompanied by a researcher. The participants were told that they would have to retell the story to the researcher later, so they were advised to be attentive and not to ask any questions as they watched the film on a laptop computer. Immediately after viewing the film, they were requested to retell the story to the researcher while being recorded on video. The researcher used the verbal prompt “*Did you like the film?*”, followed by “*Tell me about it*,” to start eliciting the narration, which was allowed to develop naturally with no further prompting. However, when the researcher felt that the storytelling failed to progress, she encouraged the participant to continue by asking unspecific open-ended questions (e.g., “*What happened then?*”).

Children’s narrative features are expected to differ depending on the type of task in which the narrative is elicited. Namely, narrative genre (fictional vs. personal) has been proven to influence the frequency of use of evaluative devices (Shiro, 2003). Fictional narratives have been elicited through different tasks and modalities [i.e., written, oral, or visual sources such as a film, single picture, comic strip, or picture book like the previously mentioned “Frog story” (Berman and Slobin, 1994)]. Some studies suggest that elicitation from oral narratives has a greater impact on the episodic structure of the retelling, while elicitation from audiovisual narratives may enhance the linguistic features of the narratives. Moreover, results seem to vary not only as a function of modality, but of elicitation procedures. If prompts are introduced, the episodic structure of the retell might be richer and better organized, but the narratives appear to be less detailed and with less syntactic complexity and lexical diversity (Gazella and Stockman, 2003).

Concerning the visual modality, the differences between elicitation methods based on static pictures vs. films have been discussed. Beyond the “Frog story” task, which mirrors an interactive book-reading format, elicitation tasks based on films have also been used, assuming that fictional stories from TV programs are the most frequent fictional narratives in the everyday lives of children and adults (Shiro, 2003). Video stories portray dynamic relationships among characters, events, and scenarios, much as in real events, so the child does not need to generate them from non-moving pictures (Gazella and Stockman, 2003). Based on a picture book of the “Frog story” series, the silent film “Frog goes to dinner” has been used in previous research to elicit narratives and assess their causal coherence and syntactic complexity in pre-school and school-age children with low and average school achievement (Gutierrez-Clellen and Iglesias, 1992; Gutierrez-Clellen, 1998).

In a recent study, we used the same film to elicit narratives from adults with WS for analysis of narrative coherence and cohesion (Diez-Itza et al., 2016). However, we considered it was too complex for the purposes of the present study as it includes children with WS in the early school years, and we found it more convenient to elicit the narratives from the Tom and Jerry cartoon. Using this method, very young TD children (3-year-olds) were able to understand the film and to generate basic oral stories after viewing it (Diez-Itza et al., 2001). Thus, we considered that it would be a feasible elicitation method in order to assess the narrative skills of individuals with limited cognitive and linguistic abilities, such as the students with WS in the present study. It also may allow for cross-syndrome comparisons and for comparisons of populations with developmental disorders to typically developing children, avoiding floor effects.

#### Transcription and Coding

The 16 video-recorded narratives were transcribed and coded using the CHAT format provided by the CHILDES Project (MacWhinney, 2000). Transcription was conducted by four trained researchers. In the first stage, each researcher transcribed 4 recordings from pretest or posttest, signaling all the unclear passages. In the second stage, each researcher revised the remaining four transcripts from pretest or posttest. In the third stage, a senior researcher resolved the final difficulties in the transcripts in order to achieve the highest agreement. Coding for microstructure and macrostructure measures was conducted in a different way. In the first stage, one of the authors coded pretest transcripts and another one coded posttest transcripts. In the second stage, the first author and the last author jointly revised the whole set of coding transcripts until total agreement was reached.

### Measures

Analysis of oral narratives is recognized as an “ecologically valid” assessment method sensitive to differences in children’s language proficiency, which has demonstrated criterion validity with standardized language measures (Tilstra and McMaster, 2007). Multiple discrete language measures at both levels, the microstructure (sentence level) and the macrostructure (discourse level), can be analyzed from transcripts of children’s oral narratives, and have the potential to document a student’s response to academic intervention. Effects of narrative intervention for school-age children with language impairment have systematically been assessed by means of microstructure and macrostructure measures (Petersen, 2011). Microstructure aspects of narrative performance have been analyzed considering productivity (lexical and utterance output) and complexity (MLU and complex syntax) (Justice et al., 2006). A number of rubrics, schemes, protocols, indexes and standardized scales have been used as outcome indicators of the effect of contextualized intervention on macrostructure productivity (elements of the story grammar) and complexity (episodic structure) (Gillam et al., 2012).

In the present study, narratives were assessed for microstructure and macrostructure, including the following productivity and complexity measures: (i) Microstructure productivity (length of narratives): Total number of utterances, total number of clauses, and total number of words (tokens); (ii) Microstructure complexity (syntactic complexity, lexical diversity and cohesion): Mean length of utterances in words (MLUw), total number of different words (types), and total number of discourse markers (cohesive devices); (iii) Macrostructure productivity (completeness of narratives): Total number of scenarios, total number of episodes, total number of events, and total number of characters; and (iv) Macrostructure complexity measures (sequential order): Order of scenes, order of episodes, order of events, and order (adequacy) of reference to characters.

#### Microstructure Measures

The microstructure measures were computed by means of the CLAN software provided by the CHILDES Project (MacWhinney, 2000). Counts of Utterances were obtained directly from the transcripts, as these are the units for transcription of the main tiers in the CHAT format. Counts of Clauses required additional segmentation coding. Clauses were analyzed as segments containing at least a finite verb or a non-finite verb (i.e., infinitive, participle, or gerund), although some clauses could contain more than a verb if one of them was a modal or an auxiliary verb. Utterances in which the verb was elliptic were also computed as a clause. Thus, some utterances may consist of a single clause (with or without a verb) while others may contain a main clause and its dependent clauses (with one or more verbs). Counts of word tokens and word types were obtained directly from the transcripts as an output from CLAN software, as well as MLUw, which is derived from productivity measures (tokens/utterances). Counts of discourse markers required additional coding of these cohesive devices. Discourse markers signal an interpretive relationship between the utterance they introduce and the prior segment in discourse. Their cohesive role at the discourse level is different from their syntactic role at the sentence level, so their more specific interpretation is given by the context (Halliday and Hasan, 1976; Fraser, 1999). Thus, in coding for discourse markers, conjunctions, adverbs, verbs, or even interjections and phrases were included when serving particular textual pragmatic functions. Discourse markers comprised progression markers, serving functions of starting, continuing, adding new information, or closing the story (e.g., *there was, and, then, that’s the end*), and interaction markers, accomplishing functions such as assertion, negation, causality, or restriction (e.g., *yes, no, because*, *but*) of what has been previously said in the dialogical parts of the narratives.

#### Macrostructure Measures

The narratives generated by the participants were compared to a complete version of the story built-up by the researchers, which served as the “gold standard” scheme for coding (see **Table 1**).

**Table 1:** “Gold standard” scheme for macrostructure coding.

SCN	EPS	EVT
1 River	1	(1) Car/bridge/bag/fall/river
	2	(2) Mouse/rescue/bag/puppy
		(3) Puppy/play/bark/lick/mouse/throw stick
		(4) Puppy/fall/mouse/rescue
2 Cat’s house	3	(5) Mouse/takes puppy/house
		(6) Puppy/can’t go into mouse’s house
		(7) Mouse/puppy/go into cat’s house
	4	(8) Puppy/lick/cat’s milk/mouse/hide puppy/cat/angry
		(9) Cat/throw puppy out house
	5	(10) Mouse/put puppy back into house/hide puppy/drawer
		(11) Puppy/get into cat’s bed/take blanket/cat/wake up/sneeze
		(12) Puppy/lick/cat/throw puppy out again/fall into a bottle
	6	(13) Mouse/puppy back into house again/puppy/lick mouse and cat
		(14) Cat/pursue/puppy and mouse
		(15) Cat/put bar of soap on floor/mouse and puppy/slide/out of house
	7	(16) Cat/sleep/storm
		(17) Thunders/cat/wake up/worried about puppy and mouse
3 River	8	(18) Cat/look for/puppy and mouse/hat/umbrella/whistle/wake up
		(19) Wind/cat/bridge/fall into the water
	9	(20) Puppy and mouse/rescue/cat/unconscious
4 Cat’s house	10	(21) Puppy and mouse/take care/cat/heat/soup/fireplace
		(22) Puppy and mouse/give soup/cat/funnel/puppy/lick/cat/wake up
		(23) Cat/give/puppy/bowl of milk/bed
		(24) Puppy/call/brothers/come running
		(25) Puppies/lick/milk/cat and mouse/look at them/happy


Narrative macrostructure was assessed based on the “Pragmatic Evaluation Protocol for the analysis of oral Corpora” (PREP-CORP), which has been used in our previous research with WS and DS groups (Fernández-Urquiza et al., 2016; Shiro et al., 2016). PREP-CORP allowed for coding of the narrative structure at three levels: (i) *Scenarios*: basic or general level, corresponding to the locations or spaces in which the initiating event, complication, high point, and resolution of the story took place; (ii) *Episodes*: intermediate or integrated level, corresponding to sets of actions whose sequencing constitute the plot of the story; (iii) *Events*: complex or detailed level, corresponding to the sequence of single actions making up the story. A total of 4 scenes, 10 episodes, and 25 events were identified in the “gold standard” version of the story.

Macrostructure productivity was assessed as the proportion (in percentage) of scenarios, episodes, and events related in the narratives of participants to the total number of them in the “gold standard” version. The reference to an event in a narrative was computed whenever an action was verbally mentioned by means of a clause, at minimum. Credit for the production of any given event was awarded to the participant based on semantic-pragmatic criteria and independently from phonological or morphosyntactic correctness. At the same time, the event was linked to the correspondent episode and scenario of the plot, as specified in the “gold standard” version. For instance, the mention of event 8 corresponded to episode 4 and scenario 2 (see **Table 2**). Furthermore, PREP-CORP provided codes for the analysis of reference to characters. Introduction of characters as a measure of narrative productivity (completeness) referred to the adequate mention of each of the three characters (Mouse, Puppy, Cat) at least once in the story.

**Table 2:** Example of retelling of episode 4 (participant 08).

PRETEST	^∗^CHI: and (.) well (.) what else (.) and he was eating food [c].
	%cod: $SCN2:EPS4:EVT8
POSTTEST	^∗^CHI: Well (.) they pass by the kitchen [c] (.) and when they do [c] (.)
	the: [//] (.) there’s a cat [c] (.) who gets angry [c] (.) because
	they’re going to drink his [/] his milk [c].
	%cod: $SCN2:EPS4:EVT8
	^∗^INV: uhum.
	^∗^CHI: ee: (.) hmm (..) later (..) then they go out [c].
	^∗^CHI: no (.) he goes out (.) the cat because he gets angry (.)
	he takes the dog out of the house the cat [c] (.)
	he takes him out [c].
	%cod: $SCN2:EPS4:EVT9


Macrostructure complexity of the narratives was assessed considering the sequential order of scenarios, episodes, and events, as well as the adequate reference to characters. The order of events was computed as the proportion (in percentage) of events that appeared in their canonical sequential order to the total of events related. The order at the level of episodes and scenarios was calculated following the same procedure. Order of characters was computed as the proportion (in percentage) of adequate references to characters occurred in a narrative to the total of events related. Adequate references were calculated subtracting the number of inadequate references to characters (i.e., lack of mention when needed, confusion, and mention of unrelated characters) from the total number of events related.

### Intervention Delivery

Explicit oral narrative intervention was delivered individually to each participant by an expert interventionist. It was a short intervention scheduled weekly during 1 month (four sessions of approximately 20 min each). Posttest assessment was conducted 2 weeks after the last intervention session. The intervention design was based on a review of previous studies researching the effects of narrative intervention both in typically developing and language impaired students. Narrative generation and retelling have been reported to be the key common factors among all the manualized intervention methods, thus narrative intervention could be procedurally simple (Petersen, 2011). Main strategies featured in narrative intervention studies included: open opportunities for students to retell, systematic support from visual materials, immediate feedback (i.e., expansions/extensions), non-restrictive prompting, and progressive scaffolding fading to build independence. The need for explicitly teaching of linguistic complexity such as the use of specific temporal and causal markers has also been underscored (Petersen and Spencer, 2014, 2016).

A semi-manualized method of intervention was devised based on these principles. During the sessions, the participant had to generate and retell the story repeatedly with visual support and immediate scaffolding from the interventionist. A set of 25 pictures captured from the movie frames was used as the visual support. Each captured picture represented roughly one of the events in the “gold standard” version of the story. Two simplified versions of the cartoon were also video-edited: a short version covering scenarios 1 and 2, and a longer version including all the scenarios, episodes and events.

Sessions started with the retelling of the story by the student without scaffolding. Then the interventionist modeled the retelling using the set of pictures in a scripted way. In order to teach explicitly the narrative structure of the story, the intervention was organized around the sequences of actions occurring within each Scenario, highlighting the Event structure of the Episodes. The first session focused on Scenarios 1 and 2. The interventionist showed the students the set of pictures corresponding to the first scenario one by one, presenting the characters, and providing explicit target verbs for actions (i.e., *fell*, *rescued*, *entered*, *ran after*), and explicit markers (i.e., *and, then, afterward*). Then, the student had to retell the events and episodes within the scenario with the visual support of the pictures and the scaffolding of the interventionist. Explicit prompts along with extension and expansion strategies were used depending on the length and accuracy of the retelling, the correct identification of characters and actions, the adequate order of events and the use of target verbs and discourse markers. The same procedure was employed to teach the macrostructure and the microstructure within the Scenario 2. The last part of the session was devoted to the viewing of the short version of the film with the support of the pictures and with the scaffolding of the interventionist. The objective was to raise awareness of the event structure of the film based on the correspondences with the pictures. After that, the participant had to retell the story without scaffolding. The second session was devoted to scenarios 3 and 4 using the same methodology. The longer simplified version including all the scenarios was used at the end of this session. The last two sessions had the same structure but focused on the story as a whole, comprising all four scenarios and underscoring the sequential relationships within the general structure: initiating events, complicating actions, high points, and resolutions. Explicit linguistic elements were still provided by the interventionist, although prompting and scaffolding were progressively reduced to boost the highest autonomy in participants’ retelling of the story at the end of the session.

Overall, the fidelity of the implementation was judged to be satisfactory on the basis of several criteria used in previous studies of effectiveness of curriculum intervention programs, indicating that it was feasible to deliver the intervention (O’Donnell, 2008). The interventionist’s adherence to the structural components of the intervention (quality of delivery) was assured by the fact that there was only one interventionist who was also involved in the intervention design. Therefore, she had a good understanding of the objectives and of the structural components and processes of the intervention, which was manualized, assuring no major variations in its delivery. Improvements at posttest of several microstructure and macrostructure measures provide further evidence that the intervention was delivered as intended and that WS students also adhered to the structural components of the intervention (participant responsiveness). Moreover, the method based on videotapes and literal transcripts allows for an accurate monitoring of the implementation of the intervention, yielding more valid indicators of fidelity than self-reports.

### Data Analysis

The effects of intervention were evaluated using a one-group pretest–posttest quasi-experimental design. This is a non-randomized within-subjects study design, which may provide more control of the variables when the sample size is small, as in rare disorders where ethical issues of therapeutic intervention may also arise. Pretest measures provided information about what the narrative performance would have been if the intervention had not occurred. Although this precedence is an important requirement of causality, and allows for the statistical assessment of variation in the outcome, the lack of randomization fails to exclude alternative explanations, which should be discussed.

The Wilcoxon signed-rank test was used as a non-parametric more powered alternative to the paired *t*-test for differences of means before and after the intervention, because the distributions did not always approximate normality as assessed with the Shapiro–Wilk test. In addition to significance tests, estimates of the magnitude of the observed effects were calculated, as they are considered an essential outcome of empirical studies. There are different definitions of a standardized effect size, which requires a choice about the statistic providing the best summary of results. Effect sizes can be grouped into two families: *r* family (based on correlations), and *d* family (based on mean differences). To better examine inherently intra-individual effects, it is recommended to incorporate the correlation between measures. Two viewpoints determine some of the practical choices when reporting results, focusing either on generalizability regardless of the research design (i.e., between- vs. within-subjects design), or on the statistical significance of the differences drawn by the statistical test. The generalizable effect size viewpoint considers that within-subjects designs overestimate effect sizes, while the statistical significance viewpoint regards this larger effect size as a benefit of a more powerful design (Lakens, 2013).

Many texts on statistics do not mention effect sizes for common non-parametric procedures as the Wilcoxon test. G^∗^Power calculates *d*_z_, the standardized mean difference effect size for within-subjects designs, based on pre- and posttest means and standard deviations, and the correlation between measures. Kerby (2014) suggested a simple difference formula to estimate effect sizes: the *r* “matched-pairs rank-biserial correlation” equals the difference between the proportion of favorable (f) and unfavorable (u) evidence from rank sums (*r* = f - u). The proportion of favorable evidence can be also considered with this type of data as the “common language effect size” estimate, as it expresses the meaning of an effect size in the everyday language of a percentage. Thus, it may be easily interpreted as how often a score sampled from the posttest distribution will be greater than a score sampled from the pretest distribution (i.e., probability of superiority). Although *d* is recommended to generalize the impact of a treatment, *r* might be a more flexible statistic and a more ecologically valid predictor of the outcome than *d* when the sample is small. In that circumstance, a multiple perspective using both *r* and *d* has been suggested (McGrath and Meyer, 2006). Further discussion of these issues, formulas, and tables for converting between several effect size estimates (Cohen’s *d*, point biserial *r*, squared eta, probability of superiority, area under the ROC curve) can be found in Fritz et al. (2012).

In order to discuss the statistical effect sizes of the differences observed between pre- and posttest measures, three different estimated values of the size of the effect were calculated for each test: (i) *d*_z_ from G^∗^Power; (ii) *r* “matched-pairs rank-biserial correlation” calculated from Kerby (2014) simple formula; (iii) Probability of Superiority (PS): common language effect size converted from *d*_z_ following Fritz et al. (2012). Gains (in percentage) after intervention were calculated on: microstructure and macrostructure, productivity and complexity, and on a global measure of overall improvements (average combined gains). Furthermore, multiple linear regression analyses were conducted to assess predictability of scores at pretest and gains at posttest from CA, Non-verbal IQ (PIQ), and initial scores on microstructure and macrostructure measures. In spite of small size of the sample, recent studies indicate that linear regression models may require only two subjects per variable for adequate estimations (Austin and Steyerberg, 2015). The proportion of variance explained by the models was drawn from the adjusted coefficient of determination (AdjR-Squared), to correct for the effects of the small sample size, and its statistical significance was tested by ANOVA (F). Coefficients of partial correlation were also calculated to assess strength and direction of the associations. In order to compare the variability of measures (i.e., heterogeneity), a standardized measure of dispersion (Coefficient of Variation: Relative Standard Deviation) was calculated as the ratio of standard deviation to the mean, and expressed as a percentage.

## Results

All the students with WS showed a sufficient level of understanding of task requirements and accomplished the narrative task at pretest. After intervention, all of them presented gains on a global measure of overall percentage of improvement (Mean: 54%; range: 14–97). Mean percentages of gain were also calculated on overall microstructure (Mean: 69%; range: 12–178) and macrostructure (Mean: 38%; range: 5–120), as well as on overall productivity (Mean: 64%; range: 6–122) and complexity (Mean: 43%; range: 11–89), on microstructure productivity (Mean: 78%; range: 11–190) and complexity (Mean: 61%; range: 3–166), and on macrostructure productivity (Mean: 51%; range: 2–121) and complexity (Mean: 25%; range: -10–118).

**Tables 3**, **4** list scores on microstructure productivity and complexity at pretest and at posttest, percentage of gains, and results of Wilcoxon test (*Z*-values) together with estimations of the effect sizes of the differences. Results indicated statistically significant differences between pretest and posttest in all six microstructure measures. After the intervention, the students with WS generated longer and more complex stories in terms of both morphosyntactic and lexical measures. Gains ranged between 27.6% (MLUw) and 93.9% (Discourse Markers), with high effect sizes in all cases (*r* range: 0.5–1; *d*_z_ range: 0.88–1.59; *PS* range: 74–87). However, very high coefficients of variation (CV) in percentages of individual improvements were observed, ranging from 80% (Types) to 116% (MLUw).

**Table 3:** Microstructure measures of narrative productivity.

	PRE	POST	Δ%	*Z*	*p*	*r*	*d*_z_	PS
UTT	15.00 (6.63)	20.38 (3.58)	54.32 (53.24)	2.113	0.035	0.50	1.12	78
CLA	26.13 (14.45)	43.63 (19.45)	85.08 (76.77)	2.524	0.012	1	1.59	87
TOK	145.88 (88.39)	252.63 (109.25)	93.27 (75.69)	2.524	0.012	1	1.33	82


*UTT, utterances; CLA, clauses; TOK, tokens.*

**Table 4:** Microstructure measures of narrative complexity.

	PRE	POST	Δ%	*Z*	*p*	*r*	*d*_z_	PS
MLU_w_	9.50 (1.89)	12.03 (3.35)	27.58 (32.01)	2.240	0.025	0.75	0.88	74
TYP	69.25 (27.56)	106.50 (38.84)	60.20 (47.92)	2.524	0.012	1	1.24	80
MRK	18.75 (9.47)	32.88 (16.86)	93.95 (94.95)	2.240	0.025	0.75	1.01	76


*MLU_w_, mean length of utterances in words; TYP, types; MRK, markers.*

At pretest, microstructure productivity measures showed higher ranges of CV (44–61%) than complexity measures (20–51%), with the lowest dispersion observed in MLUw, and the highest in Tokens. At posttest, dispersion of productivity measures was reduced (18–45%), while it increased in complexity measures (28–51%).

**Tables 5**, **6** list scores on macrostructure productivity and complexity measures at pretest and at posttest, percentage of gains, and results of Wilcoxon test (*Z*-values) together with *r, d*_z_, and *PS* estimations of the effect sizes of the differences. Results indicated statistically significant differences between pretest and posttest in all macrostructure productivity measures, except for character introduction. After the intervention, the students with WS generated more complete stories at the integrated and detailed levels (episodes and events), and they included all the scenarios and characters. Gains ranged between 20.8% (scenarios) and 103.3% (events). Significant differences showed high effect sizes (*r* range: 0.62–1; *d*_z_ range: 1.18–2.62; *PS* range: 80–97). Again, very high coefficients of variation in percentages of individual improvements were observed, ranging from 70% (events) to 225% (characters). Conversely, no significant differences were observed in macrostructure productivity measures, which might be related both to high scores at pretest, and to the fact that as narrative productivity increases ordering difficulties grow to a similar extent. Effect sizes were near chance, except for order of characters, but improvements showed the greatest heterogeneity.

**Table 5:** Macrostructure measures of narrative productivity.

	PRE	POST	Δ%	*Z*	*p*	*r*	*d*_z_	PS
SCN	84.38% (12.94)	100% (0.00)	20.83 (17.25)	2.236	0.025	0.62	1.18	80
EPI	65% (23.91)	86.25% (10.61)	48.85 (56.65)	2.388	0.017	0.87	1.29	82
EVT	37% (18.24)	64.50% (13.93)	103.33 (72.58)	2.530	0.011	1	2.62	97
CHT	87.50% (24.87)	100% (0.00)	31.25 (70.39)	1.342	0.180	0.25	0.50	64


*SCN, scenarios; EPI, episodes; EVT, events; CHT, characters.*

**Table 6:** Macrostructure measures of narrative complexity.

	PRE	POST	Δ%	*Z*	*p*	*r*	*d*_z_	PS
SCN	91.67% (15.43)	93.75% (11.57)	4.69 (21.06)	0.535	0.593	0.12	0.13	53
EPI	88.14% (13.68)	91.52% (16.42)	6.03 (23.63)	0.674	0.500	0.37	0.16	53
EVT	89.35% (12.54)	89.55% (17.44)	1.24 (20.30)	0.314	0.753	0	0.01	50
CHT	58.09% (30.67)	75.67% (17.76)	87.43 (150.10)	1.352	0.176	0.12	0.56	66


*SCN, scenarios; EPI, episodes; EVT, events; CHT, characters.*

At pretest, macrostructure productivity measures showed ranges of CV (15–49%) similar to the ranges of dispersion of complexity measures (14–51%). The lowest coefficients of variation were observed in recall of scenarios and order of events, and the highest in recall of events and order of characters. At posttest, heterogeneity was reduced in productivity measures (0–22%), and to a lesser extent in complexity measures (12–23%).

In order to determine which measures at pretest are the best predictors of gains after intervention, multiple regression analyses were conducted controlling in each case for the respective microstructure and macrostructure productivity or complexity variables. Partial correlations indicated positive or negative direction of the relationships between variables. Participants producing a higher number of utterances showed lower gains in microstructure productivity (AdjR-Squared = 0.423; *F* = 6.130; *p* < 0.048), and lower global improvement of narratives (AdjR-Squared = 0.545; *F* = 9.368; *p* < 0.022). Number of types and discourse markers jointly predicted gains in macrostructure productivity (AdjR-Squared = 0.598; *F* = 6.213; *p* < 0.044): participants with more cohesive narratives (in Discourse Markers) but in proportion less lexical diversity tended to show higher gains in recall of macrostructure. Recall of scenarios, episodes and characters jointly predicted gains in macrostructure productivity (AdjR-Squared = 0.978; *F* = 106.874; *p* < 0.001), macrostructure complexity (AdjR-Squared = 0.716; *F* = 6.882; *p* < 0.047), and overall macrostructure (together with events) (AdjR-Squared = 0.967; *F* = 51.934; *p* < 0.004): gains in productivity were positively predicted by scenarios, and negatively by episodes and characters, while gains in complexity were positively predicted by episodes, and negatively by scenarios and characters, and gains in overall macrostructure showed the same directions of associations and, in addition, a negative one with events. Order of scenarios, episodes, events and characters predicted gains in macrostructure complexity (AdjR-Squared = 0.955; *F* = 38.414; *p* < 0.007): participants with higher order in events but in proportion lower order of scenarios, episodes and characters showed higher gains.

In order to estimate linear dependence between CA or non-verbal IQ (PIQ) and performance at pretest and posttest and gains, multiple regression analyses were conducted, controlling in each case for the respective microstructure and macrostructure productivity or complexity variables. Partial correlations indicated positive or negative direction of the relationships between variables. At pretest, CA significantly predicted utterances, discourse markers, MLUw, and events and characters recalled. At posttest, CA only predicted events recalled, and order of scenarios and characters. Furthermore, CA predicted gains in utterances and in episodes, events and characters recalled. At pretest, non-verbal IQ (PIQ) predicted scenarios, episodes and characters recalled. At posttest, PIQ predicted order of these same variables, and also MLUw and discourse markers. Furthermore, PIQ predicted gains in events and characters recalled and in order of events.

At pretest, older participants generated longer narratives (in utterances) (AdjR-Squared = 0.567; *F* = 10.151; *p* < 0.019), and more cohesive (in discourse markers) but in proportion less complex ones (in MLUw) (AdjR-Squared = 0.775; *F* = 13.090; *p* < 0.010). CA also predicted jointly events and characters recalled before intervention (AdjR-Squared = 0.952; *F* = 70.748; *p* < 0.001): older participants generated more complete narratives (in events), but they included in proportion less characters. At posttest, older participants still generated more complete narratives in terms of events recalled (AdjR-Squared = 0.481; *F* = 7.490; *p* < 0.034), and also more ordered ones at the level of scenarios and characters (AdjR-Squared = 0.665; *F* = 7.935; *p* < 0.028). Percentage of gain in utterances was higher in younger participants (AdjR-Squared = 0.475; *F* = 7.331; *p* < 0.035). CA also predicted jointly percentage of gain in events, episodes and characters recalled (AdjR-Squared = 0.708; *F* = 6.655; *p* < 0.049): younger participants presented with more gains in events recalled, but in proportion their gains in episodes and characters were lower.

At pretest, PIQ predicted jointly scenarios, episodes and characters recalled (AdjR-Squared = 0.877; *F* = 17.628; *p* < 0.009): participants with higher PIQ recalled more episodes, but they included in proportion less Scenarios and Characters. At posttest, participants with higher PIQ produced longer utterances (in MLUw) but in proportion their narratives were less cohesive (in discourse markers) (AdjR-Squared = 0.742; *F* = 11.090; *p* < 0.015). PIQ also predicted jointly order of scenarios, episodes and characters after intervention (AdjR-Squared = 0.776; *F* = 9.101; *p* < 0.029): participants with higher PIQ showed more order in scenarios and characters, but in proportion less order in episodes. PIQ predicted jointly percentage of gain in events and characters recalled (AdjR-Squared = 0.767; *F* = 12.539; *p* < 0.011): participants with lower PIQ showed more gains in events recalled, but in proportion their gains in recall of characters were lower. Participants with higher PIQ showed higher improvements in order of events recalled (AdjR-Squared = 0.453; *F* = 6.792; *p* < 0.040).

## Discussion

The aim of the present study was to determine the feasibility and possible effects of oral narrative assessment and intervention for students with WS. In the case of students with developmental disabilities, pragmatic narrative competence might be essential for school inclusion and achievement, as it provides a crucial bridge between linguistic abilities and cognitive and social skills. Narrative-retelling and narrative-generation tasks constitute a natural, appropriate context for the dynamic assessment of pragmatic abilities from the early years and throughout the school age. They have been used repeatedly in the research on pragmatic abilities of students with developmental disorders and language impairment. However, to our knowledge, no research of narrative intervention for individuals with WS had so far been conducted. Fictional narratives in the present study were elicited from an episode of the “Tom and Jerry” cartoon series at pre- and post-intervention sessions, and they were transcribed and coded for microstructure and macrostructure analyses at sentence- and discourse-levels. The analyses at the microstructure level included measures of productivity (utterances, clauses and words) and complexity (MLUw, lexical diversity and use of discourse markers). The analyses at the macrostructure level included measures of productivity and complexity (story completeness and sequential order in terms of scenarios, episodes, events and characters).

At pretest, all the students with WS showed, at minimum, basic abilities to autonomously generate narratives about some of the characters and events presented in the film. This is consistent with previous results from 3-year-old typically developing preschoolers and DS MLU-matched children using the same elicitation task (Diez-Itza et al., 2001; Fernández-Urquiza et al., 2016). Consequently, no floor effects showed for any of the measures, although a high variability in narrative proficiency within WS students both at microstructure and macrostructure levels was observed. While younger participants performed near floor, some of the older generated quite complete and ordered narratives, which might have had a ceiling effect on intervention outcomes.

Therefore, the method could be adequate for narrative assessment at very early stages of linguistic and cognitive development, but in the case of older students with WS, a more complex story would possibly allow larger room for improvement. In a previous study of young adults with WS, where the narratives were elicited from a more complex story, the scores were higher than those obtained by the students in the present study, which may also be explained by the fact that participants were older and showed higher levels of cognitive and linguistic development (Diez-Itza et al., 2016).

After intervention, all the participants showed overall improvement in a global measure of narrative performance. The best outcomes were observed at the microstructure level, with higher improvements in productivity (i.e., story length). Gains in macrostructure productivity (i.e., story completeness) paralleled overall improvement, but no significant gains were observed at the macrostructure complexity level (i.e., story order). At posttest, WS students generated narratives with more utterances, which included more clauses and tokens. The length of the utterances and the lexical diversity also increased. The highest gains were observed in the use of discourse markers, which were explicitly taught in the intervention sessions to enhance narrative cohesion.

Improvements in language productivity and complexity allowed the students with WS to generate more complete narratives, achieving the highest advances in event recall. Their stories showed considerably more detail after intervention, which could indicate that extensions at the microstructure level can be reflected in narrative macrostructure. Moreover, when controlling for lexical diversity, the participants with more cohesive narratives at pretest had better outcomes. Therefore, the use of discourse markers might be a good predictor of narrative development in school-age children with WS. Narrative integration at the level of episodic structure also showed significant improvement, but it did not parallel the gains in event detail. This proclivity to recall details should be taken into account in future intervention designs, as it could generate an imbalance between over-detailed and under-detailed or omitted episodes within narrative structure.

In a previous study, we had already observed the relative disproportion between event recall and episode integration in narratives of adults with WS (Diez-Itza et al., 2016). The tendency of WS individuals to focus on elaborated descriptions of episodes, weakening the thematic structure of narratives, had also been reported and was interpreted in terms of a dissociation between linguistic abilities and pragmatic integration skills (Reilly et al., 2004). Lack of integration has been related to a detail-focused processing style that is also observed in individuals with ASD (Happé and Frith, 2006). Children with ASD share with their WS pairs a relative weakness in narrative macrostructure, and their stories have been considered simplistic when analyzed for macrostructure features such as organization and causal coherence (Capps et al., 2000; Norbury et al., 2014; Gillam et al., 2015). Children with WS also presented narratives with fewer components and lower integration of thematic structure and characters than SLI pairs, which was discussed in terms of the role of general cognitive impairment (Reilly et al., 2004).

However, syndrome-specific differences in cognitive processing should also be considered, as children with DS and FXS may develop relative strengths in narrative macrostructure (Boudreau and Chapman, 2000; Finestack et al., 2012; Channell et al., 2015). Cognitive impairments in areas such as spatial cognition might account for those differences. In construction tasks, WS individuals present a tendency for local processing and a difficulty in perceiving global structure, which has been explained as an interactive effect of faulty executive processes and fragile spatial representations (Mervis, 2006). In a previous study, we suggested a possible relation between weaknesses in narrative construction and deficits in global processing, but we failed to find a significant correlation between measures of the Block Design subtest of the Wechsler Intelligence Scales and measures of narrative structure and sequential order (Diez-Itza et al., 2016). Individuals with WS showed more difficulties in macrostructural processing of narratives in a picture-sequence task than in a single picture task, which was discussed as related to deficits in sequential analysis and spatial working memory (Marini et al., 2010). Nevertheless, a direct link between measures of attention or visual-spatial skills and narrative processing was not found, so the authors pointed out that the story effect could be due to the higher narrative skills required to generate a story from a sequence of pictures. Specific research would be needed to better assess the hypothesis of a relationship between cognitive spatial and textual pragmatic domains.

The present study failed to evidence improvements in character introduction, which may be related to near ceiling scores at pretest, as the majority of participants had initially introduced all of the characters. Limited computing for character appearances might also account for this difference. Furthermore, the students with WS did not show advances in macrostructure complexity (i.e., sequential order of events and adequate character management). This could be similarly explained by high-ordered stories at pretest and moreover, by the fact that order keeps a proportion to the total number of scenarios, episodes, events and recalled characters. Increased length of narratives entails greater difficulties in maintaining canonical order of the events, episodes and scenarios. Therefore, future intervention designs should put more focus on macrostructure organization, as the current results confirm that individuals with WS persistently struggle with building narrative coherence and thematic structure. This is consistent with findings of previous research in different languages (Reilly et al., 2004; Garayzábal Heinze et al., 2007; Lacroix et al., 2007; Gonçalves et al., 2010; Marini et al., 2010; Diez-Itza et al., 2016).

Special difficulties with character management were found prominent, including lack of mention when needed, confusion, and mention of unrelated characters, and they continued to be the weakest aspect of narrative performance after intervention. It must be acknowledged that the narrative intervention design of the present study lacked a sufficient and explicit focus on such specific problems, although they had been suggested by some prior research. Reilly et al. (2004) reported failure to integrate characters in the thematic structure of the stories as a consequence of intellectual impairment. In a previous study, we found that children with DS showed verbal-age levels in macrostructure levels, but they performed under verbal-age in adequate reference to characters (Fernández-Urquiza et al., 2016).

As expected given the wide range of ages of participants, performance at pretest and gains after intervention could be in part predicted by CA. At pretest, older students with WS generated narratives that were longer, more cohesive, and more complete. Conversely, younger participants showed more gains in story length and completeness after intervention, while older students tended to show more improvement in episodic organization and character management. Different benefits of intervention with age could be partially explained by increase in IQ as reported by a longitudinal study of students with WS from age 12 to age 21 (Udwin et al., 1996). In line with this, a strong correlation was found between non-verbal IQ (PIQ) and CA. Nevertheless, PIQ was a predictor only of performance on macrostructure, with the exception of a positive relation between PIQ and MLUw after intervention (i.e., MLUw reached non-verbal IQ levels). Students with higher PIQ scores showed better episode integration at pretest and greater gains in the ordering of events. Conversely, students with lower PIQ exhibited higher improvements in event detail. These results support the idea that relation exists between specific features of cognitive processing and narrative coherence in WS, which could be quite independent from linguistic productivity (Reilly et al., 2004).

Losh et al. (2000), also using regression analyses, found that WS children performed at non-verbal mental age levels in the “Frog story.” They reported that CA had effects in increasing the length of narratives but not in reducing morphological errors. In previous studies, we also observed the independence of morphological errors from verbal and CA in spontaneous speech (Diez-Itza et al., 2017), but individuals with WS scored at verbal-age in narrative productivity (Shiro et al., 2016). Similar results concerning the length of the stories in number of propositions and utterances had been already reported for English-speaking and French-speaking school-age children where the stories of WS participants were longer than those of DS controls but comparable to mental-age matched TD controls (Reilly et al., 1990; Lacroix et al., 2007).

The relationships between performance at pretest and outcomes after intervention were also assessed in the present study. Participants with shorter stories (in utterances) showed higher gains in microstructure productivity and, most importantly, in overall narrative performance. Macrostructure productivity was predicted by greater use of discourse markers when controlling for number of types. Episode integration was related to higher gains in narrative complexity and lower gains in narrative productivity and overall macrostructure. Finally, higher order of events and lower order of episodes predicted gains in macrostructure complexity.

Number of utterances and use of discourse markers as measures of length and cohesion of narratives may be considered more accurate and predictive when it comes to assess narrative productivity. Conversely, MLUw as a measure of grammatical complexity demonstrated lower sensitivity and predictivity of narrative skills. MLU in morphemes ranging 1–4.4 had proven to be a reliable measure of language development in natural conversational settings as reported by Levy and Eilam (2013) in a longitudinal study with Hebrew-speaking children with DS and WS (under 8 years old) and a TD group (under 4 years old). The authors found high correlations between MLU and most morphosyntactic and vocabulary variables, and high intercorrelation between linguistic variables within MLU stages. Differences in the task (conversational vs. narrative), age of participants (above 8 years old), and MLU values (above MLU 5 in words) may account for the lack of association between MLU and linguistic measures of narrative productivity in the present study. However, our study failed to sufficiently account for grammatical complexity of the narratives and more in-depth analyses would be required for a better assessment of narrative production at the grammar level.

Inter-individual differences are more salient in populations with developmental disabilities, and the present study revealed high levels of variability in microstructure and macrostructure measures at pretest, as well as in the outcomes. It is important to note that beyond the search for syndrome-specific patterns and homogeneous profiles in neurodevelopmental disorders, the focus on group means and similarities rather than individual differences has been challenged. Porter and Coltheart (2005) questioned methodological limitations of studies of the WS cognitive and developmental profiles based on chronological and mental-age control groups and standardized instruments. They claimed that research focusing on specific task performance and group means tends to hide individual variability and they found no evidence of homogeneous strengths and weaknesses in WS. Notably, their results were inconsistent with the claim for strengths in verbal abilities.

Heterogeneity in cognitive and linguistic abilities of the students with WS in the present research could then account for the high variability of narrative performance at pretest and of individual improvements after intervention. However, the sample size is too small to discuss with more detail the sources of within group variability, and further analysis would be needed in order to better assess the differences observed. Cluster analyses may be adequate tools for assessing the distances between individuals and determine possible subgroups and extreme cases. Preliminary evidence for homogeneous subgroups in different cognitive measures was also reported by Porter and Coltheart (2005). Based on a smaller sample and on standardized and conversational linguistic measures, Stojanovik et al. (2006) found striking individual differences in all linguistic measures, which were interpreted in terms of a heterogeneous linguistic profile in WS. These authors also suggest the need for research on subgroups within WS. Determining whether or not subgroups based on narrative proficiency measures might correspond to different stages in narrative development, such as the three-phase model (preschoolers, schoolchildren, and adults) described by Berman (1988), would provide useful information to better address intervention strategies. Results from the present study show outstanding evidence of differential responses from each of the students with WS to the challenges of narrative generation and to intervention, beyond the above-discussed variability due to age and non-verbal IQ.

It is important to acknowledge several limitations of the present study. First, the design tells us about improvements of students with WS regarding several measures of narrative productivity and complexity following the intervention, but it does not allow to establish a causal relationship between intervention and outcomes. It also does not tell us whether the students would have improved regardless of the intervention, as measurements at pretest and posttest may have varied due to random error and to the regression to the mean effect. Although effect sizes of differences after the intervention were strong, they may have been overestimated by the regression to the mean effect. Second, the design does not tell us whether another approach would have been more effective. Narrative intervention is still at an emerging state of evidence, and a general focus on effectiveness has prevailed over a more precise account of the diverse intervention methodologies. The pilot intervention devised in the present study may be considered too short, but it was intended only as a preliminary design to assess the feasibility of narrative intervention for students with WS. Only a few studies have discussed about the elements of the intervention design, such as group size and intensity of intervention. A series of studies using the *Story Champ* intervention curriculum allowed for a discussion of arrangements or tiers of intervention (large-group, small-group, and individual), as well as of frequency and duration of the sessions. Individual intervention was considered the most intensive arrangement, and it provided better outcomes in spite of shorter less-intense sessions of 10–15 min (Spencer and Slocum, 2010; Petersen et al., 2014; Spencer et al., 2015). Third, although there was a 2 week lapse between the last intervention session and posttest, which may indicate a mid-term maintenance of the effects, a long-term follow-up would be necessary to assess more distal outcomes. Fourth, the present study did not include probes of generalization of outcomes to new fictional stories or to different genres. Retelling of fictional stories may facilitate the kind of historical support described by Ninio and Snow (1996), but narrative intervention should also include activities to promote transfer of learning to narratives of personal experience (Petersen and Spencer, 2016). Personal-themed social stories introduced in the natural school environment have been found to improve social behavior in students with ASD (Scattone et al., 2006). However, additional research is needed to assess effectiveness of narrative intervention in natural settings, for the evidence of proficient storytelling as related to improving opportunities for interaction and social engagement of individuals with language impairment and developmental disabilities remains indirect. Fifth, previous research on narrative intervention was conducted in many cases with small samples, but they were more homogeneous that the sample investigated in the present study. The age range of the students with WS was too broad to avoid effects of age and changing trajectories of development. Such an extended age span allowed for a broader exploration of the feasibility of explicit oral narrative intervention for students with WS at different school settings and levels. However, further in-depth case analyses should be conducted to better account for heterogeneity and differences in the outcomes. Sixth, the narrative task avoided floor effects at pretest, but some of the students with WS accomplished the task with high scores, which left them with less room for improvement. This near ceiling effect could partly explain the reduction of variability at posttest, although only macrostructure measures of scenarios and characters reached ceiling in some cases. Furthermore, the aim of the intervention was to train the students to accomplish the narrative task successfully and, consequently, to promote errorless learning, which entailed an inherent ceiling effect. Therefore, it is not a question of merely using instead a longer and more complex task, but of adjusting assessment and intervention designs to provide different levels of difficulty and scaffolding. In fact, shorter stories can provide similar reliability and sensitivity than longer ones when the design and the scoring systems are appropriate (Spencer et al., 2013; Petersen and Spencer, 2014). Finer-grained measures of grammatical complexity, discourse cohesion and episodic structure would also be needed to better assess the effect of narrative intervention.

## Conclusion

Despite substantial limitations, this study extends previous research on both narrative intervention and WS by demonstrating the feasibility and possible effectiveness of a short oral narrative intervention in enhancing pragmatic skills of students with WS. Explicit narrative intervention has been proposed as a flexible and valid framework for language assessment and intervention in natural school settings, which has the potential to foster the development of language and social skills necessary to prevent school failure. However, only a few studies evaluating narrative intervention have included students with developmental disabilities. Therefore, it may be introduced as a novel intervention technique to improve cognitive and social functioning in students with WS, which may draw on their best linguistic and social abilities. Building on strengths to optimize the potential for growth has been considered a high priority of intervention programs for children with WS (Semel and Rosner, 2003). However, the remarkable language skills of school-age children with WS have frequently led to a misperception of their needs in this area, and language intervention has been omitted or discontinued (Mervis and Velleman, 2011). The results of the present study confirm that WS individuals could benefit from language intervention despite language production being considered a relative strength in this population. After intervention, younger students with lower PIQ who at pretest generated shorter stories tended to show greater gains, above all in microstructure and macrostructure productivity, while older students improved narrative complexity to a greater extent. Interventions for pragmatic language use and social conversational skills necessary to tell coherent narratives may usefully become part of the educational profile of students with WS. Narratives are natural language samples that very closely reflect the linguistic abilities children are required to master both for social interaction at school and academic achievement. As long as narrative intervention enhances storytelling proficiency it may give students with WS more opportunities to practice language in school contexts and to get more attention and rewards from the social environment. Since this is a pilot study, further research is needed to validate the feasibility of narrative intervention for school-age children with WS. Ultimately, it is essential to bridge the gap between research and implementation of evidence-based contextualized intervention for students with WS at risk of school failure.

## Ethics Statement

This study was carried out in accordance with the recommendations of the “Red de Comités de Ética de Universidades y Organismos Públicos de Investigación de España” with written informed consent from all the legal tutors of the subjects. All of them gave written informed consent in accordance with the Declaration of Helsinki. The protocol was approved by the “Comité de Ética en la Investigación de la Universidad de Oviedo”.

## Author Contributions

ED-I had a primary role in the conception and design of the study, in the development of the coding scheme, in data analysis and discussion and in drafting the manuscript. VM helped with the design of the intervention and conducted it, carried out transcription, coding and data analyses, and helped draft the manuscript. VP assisted with transcription, coding and data analysis, and manuscript revision. MF-U had a primary role in the development of the coding scheme, conceptualization of variables, transcription and coding, and drafting the manuscript. All authors have read and approved the final version of this manuscript.

## Conflict of Interest Statement

The authors declare that the research was conducted in the absence of any commercial or financial relationships that could be construed as a potential conflict of interest. The handling editor declared a shared affiliation, though no other collaboration, with several of the authors, ED-I, VM, and MF-U.
